# RETRACTION: Rehabilitation Training and Resveratrol Improve the Recovery of Neurological and Motor Function in Rats after Cerebral Ischemic Injury through the Sirt1 Signaling Pathway

**DOI:** 10.1155/bmri/9851861

**Published:** 2026-07-03

**Authors:** BioMed Research International

RETRACTION: N. Shi, C. Zhu, L. Li, “Rehabilitation Training and Resveratrol Improve the Recovery of Neurological and Motor Function in Rats after Cerebral Ischemic Injury through the Sirt1 Signaling Pathway,” *BioMed Research International*, 2016, 1732163, https://doi.org/10.1155/2016/1732163

The above article, published online on 27 December 2016 in Wiley Online Library (wileyonlinelibrary.com), and its correction (https://onlinelibrary.wiley.com/doi/10.1155/2017/1532462), has been retracted by agreement between the journal Editor‐in‐Chief, Yoel Klug; and John Wiley & Sons Ltd. The retraction has been agreed due to concerns raised regarding the Figures, initially reported on PubPeer [1]. Further investigation by the publisher identified additional concerns with multiple figures, specifically:

• In Figure 1, there is an unexpected, repeated region between panels C, D and E
• In Figure 1, panels D and E appear identical
• In Figure 1:
  o. Panel A appears identical to the Sham Operation panel in Figure 1a of [2]
  o. Panel B appears identical to the Sham Operation panel in Figure 2a of [2]
  o. Panel C appears identical to the ZPP panel in Figure 2a of [2]
  o. Panels D and E appear identical to the ZPP panel in Figure 1a of [2]
• In Figure 2, there is an unexpected, repeated region between panels C and D
• In Figure 2, panels A and B appear identical
• In Figure 2, panel E appears identical to the ICH panel in Figure 2a of [2]
• In Figure 3a, the first and second BDNF bands appear identical
• In Figure 4a, the TrkB bands appear highly similar to the left‐side Sirt1 bands shown in Figure 2 of [3]
• In Figure 4a, the GAPDH bands appear highly similar to the left‐side GAPDH bands shown in Figure 2 of [3]

The authors did not respond to an inquiry from the publisher, and after assessment by the Editorial Board the results and conclusions can no longer be considered reliable. The article is therefore retracted.

The authors were unresponsive when informed about the retraction.
